# Spiritual Needs of Older Adults Living with Dementia: An Integrative Review

**DOI:** 10.3390/healthcare11091319

**Published:** 2023-05-04

**Authors:** Katherine Carroll Britt, Augustine C. O. Boateng, Hui Zhao, Francesca C. Ezeokonkwo, Chad Federwitz, Fayron Epps

**Affiliations:** 1 NewCourtland Center for Transitions and Health, Department of Biobehavioral Health Sciences, School of Nursing, University of Pennsylvania, Philadelphia, PA 19104, USA; 2 Department of Biobehavioral Health Sciences, School of Nursing, University of Pennsylvania, Philadelphia, PA 19104, USA; 3 School of Nursing, James Madison University, Harrisonburg, VA 22807, USA; 4 Gerontology, Western Colorado Community College, Grand Junction, CO 81505, USA; 5 Nell Hodgson Woodruff School of Nursing, Emory University, Atlanta, GA 30322, USA

**Keywords:** Alzheimer’s, spiritual care, spiritual support, successful aging, unmet needs, meaning, connectedness, peace

## Abstract

Older adults living with dementia experience progressive decline, prompting reliance on others for spiritual care and support. Despite a growing interest in studying persons living with dementia (PLwDs), empirical evidence on the spiritual needs of PLwDs has not been synthesized. Using the Whittemore and Knafl method, this integrative review examined the literature from 2000 to 2022 on the spiritual care needs of PLwDs. We sought to identify characteristics of the spiritual needs of PLwDs and ways to address them. The ATLA Religion, CINAHL, PsycINFO, PubMed, and Socindex databases were used to search the literature, and 12 peer-reviewed articles met the inclusion criteria. Spiritual care needs varied across studies. Overall, findings support the importance of identifying PLwDs’ religious and spiritual backgrounds to inform person-centered care. Spiritual needs were identified as verbal and non-verbal expressions related to past meaning and religious and spiritual background and were not consistently addressed in care. Providers reported observing spiritual distress in the mild stage prompting the need for spiritual care. There is a great need for dementia-specific spiritual assessment tools and spiritual care interventions to support spiritual well-being in dementia care. Spiritual care involves facilitating religious rituals and providing spiritual group therapy and religious and spiritual activities.

## 1. Introduction

Dementia results from primary and secondary effects on the brain from various diseases and injuries such as Alzheimer’s disease or stroke [1]. One of the significant causes of disability among older adults, dementia is the seventh leading cause of death among all diseases and has been declared a global public health priority [1]. Currently, more than 55 million people aged 65 or older are living with dementia. By 2030, this number is projected to rise to 78 million [2]. PLwDs show a wide range of decline in memory, thinking, and behavioral performance [1]. In addition, caregivers of PLwDs often carry out multiple tasks while caring for their care recipients. Therefore, some essential aspects of care, such as spiritual needs, can be easily overlooked [3] or difficult to identify [4,5], especially in the later stage of dementia.

Spirituality is vital in promoting health and well-being [6], providing structure, meaning, and understanding through daily life [7,8]. Studies report the potential benefits of spirituality for older adults, such as maintaining social interaction and health, developing coping strategies, fostering personhood, recovery from illness, and promoting positive aging [9,10,11]; spirituality is also associated with decreased levels of psychological stress, including depression [9,12,13,14]. Defined by an international consensus conference to coin an agreed-upon definition for spirituality through a Delphi study [15], spirituality is the way in which a person seeks and expresses meaning and purpose and experiences connectedness to the moment, self, others, nature, and the significant or sacred; spirituality is expressed through beliefs, practices, values, and traditions. Persons facing advanced illness have increased spiritual needs requiring spiritual care [4,16], a dimension of palliative care [17]. Spiritual care and support are often reported as the most neglected dimension [18,19]. Spiritual needs, like physical needs, are essential for human beings [20]. More specifically, spiritual needs encompass meaning, life purpose, and connectedness to [21,22] established beliefs, practices, values, and traditions. These characteristics are essential to spiritual care as meaning relates to what is valuable to an individual, including life choices and personal values supporting well-being [21].

In a recent literature review on spirituality and religion in dementia [23], findings support the importance of spirituality to PLwDs in finding meaning, hope, and connection to the past, present, and future and in coping with their condition [24,25,26]. PLwDs rely on others to support their spiritual well-being, particularly as their condition advances [27]. However, there is an absence or minimal presence of spiritual care in dementia care Clinical Practice Guidelines [28,29], and a lack of spiritual support at the end of life, indicating PLwDs are at risk of not having their spiritual needs met [28,30,31]. If spiritual needs and concerns are not addressed, spiritual distress may occur, which contributes to poorer patient outcomes: depression [32], diminished quality of life [33], increased anxiety, greater physical pain [34], and decreased emotional well-being [35]. Unaddressed spiritual needs can also disrupt the building of trust between healthcare providers and families [16]. In order to address the spiritual needs of PLwDs, we must identify their spiritual needs and ways to address them, especially from the perspective of PLwDs, ideally in the mild stage. However, existing studies have limited research on dementia resulting in a limited understanding of spiritual needs in this population, highlighting the need for further investigation.

To address this gap, we conducted an integrative literature review to answer the following questions: (1) what do we know about the spiritual needs of PLwDs, and (2) how are spiritual needs addressed for PLwDs?

## 2. Materials and Methods

### 2.1. Study Design and Population

**Figure 1:**
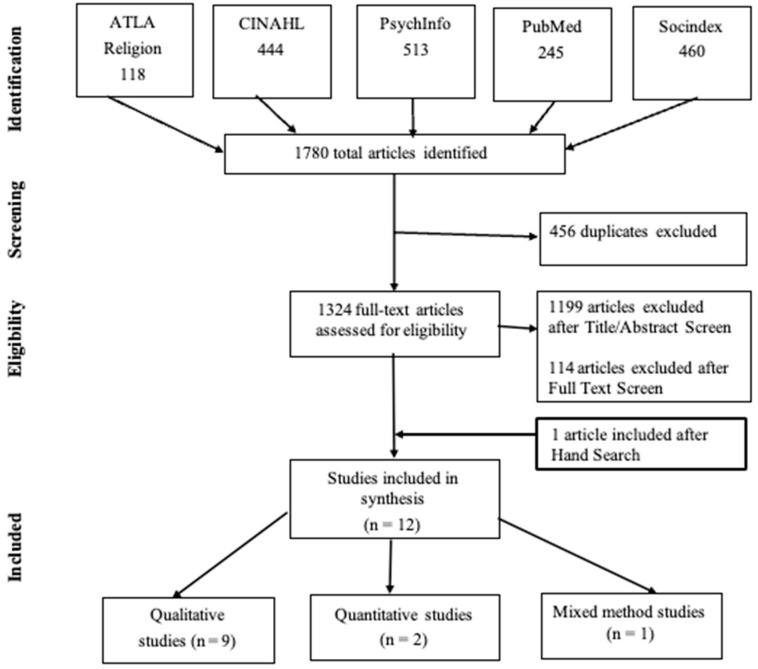
Preferred reporting items for systematic review and meta-analyses (PRISMA) selection of articles for review [37].

Using the methodology described by Whittemore and Knafl [36], this integrative review studies and summarizes previous research by drawing conclusions from studies believed to identify and address spiritual needs in PLwDs. Whittemore and Knalfl’s approach is a comprehensive and inclusive review method that enables reviewers to include studies with diverse methodologies due to variability in study purposes, designs (qualitative, quantitative, mixed), and sample characteristics. Thus, this method which comprises problem identification, literature search, data evaluation, data analysis, and presentation of conclusions, was utilized to minimize review bias and increase this study’s rigor. In addition, the Preferred Reporting Items for Systematic Reviews and Meta-Analyses (PRISMA) guidelines [37] informed our analysis and report (see Figure 1).

Spiritual needs are needs related to one’s spirituality. Terms in research, spirituality, and religion overlap and are often used interchangeably in studies [38]. For this literature review, we focused on spirituality but included papers referencing religion. However, our focus was not on religiosity, which refers to how committed a person is to their religious beliefs and principles, but on their needs for spirituality (i.e., meaning, purpose, and connectedness to their established beliefs, practices, values, and traditions). Religious activities can support one’s spiritual needs as religious institutions are designed to facilitate spirituality, supporting one’s search for significance [10].

### 2.2. Search Strategy

A literature search was conducted across five major academic databases, ATLA Religion, CINAHL, PsycINFO, PubMed, and Socindex. The development of key terms and search strategies were carried out in comprehensive collaboration with an experienced librarian in spirituality from the University of Texas Health Sciences Library. As definitions of spirituality vary, presenting empirical challenges, we chose to focus on the specific terms of ‘spiritual needs’ and ‘spiritual care’ in our search to compile studies that truly capture the concept of ‘spiritual needs’, and not just on one aspect, such as meaning [13]. After careful consideration of options and searches, the following keywords and combinations were used for the search strategy: spirituality, spiritual needs, religious needs, spiritual care, existential care, faith, dementia, Alzheimers, cognitive impairment, memory loss, Lewy body, Lewy bodies, and cognitive decline (Table 1). They were combined using the boolean operators AND and OR for electronic searches conducted in the mentioned databases. The initial database search yielded 2815 articles. Limiting the search to the years between 2000 and 2022 to focus the review on the most recent literature resulted in 2580 articles. When the search was additionally limited to English-only articles, the number of articles became 2461. Finally, 1780 articles remained when the search was limited to only peer-reviewed articles.

### 2.3. Inclusion/Exclusion Criteria

After the initial search, articles were exported into Endnote X9, where duplicates were removed. The remaining articles were then exported into Rayyan software for screening. Titles and abstracts were vetted for inclusion and exclusion criteria and independently evaluated by two of this study’s authors. When an article’s title and abstract were insufficient to make a decision, the article’s full text was retrieved and reviewed. Only papers written in English, peer-reviewed, and focusing on the spiritual needs of PLwDs were included. Articles were excluded if (1) they were not original empirical research, (2) they were literature reviews or case studies, (3) participants were nonadults (i.e., younger than 18 years of age), and (4) the concept of spirituality or spiritual care needs were not discreetly discussed (i.e., the article discussed mindfulness or meditation). Discrepancies regarding the inclusion of articles were discussed and resolved among the authors. Finally, 12 articles met the criteria and were included in this review.

### 2.4. Data Extraction

Two authors independently reviewed full-text relevant articles, extracting key information into a Word file table for the organization. A standardized data extraction form was used to guide the synthesis, with organized categories informed and created from included study characteristics, findings, and a previous spirituality and dementia literature review [39]. These included authors, year of publication, the purpose of the study, study setting, sample description, stage of dementia, study design, and major relevant findings (Table 2). The first five authors organized the data into categories. Then, the group examined and resolved any discrepancies until a consensus was reached.

### 2.5. Methodological Quality

Two authors appraised the methodological quality of included studies. The first author scored all included studies (N = 12), while the second assessed four randomly selected studies to ensure scoring reliability. The Mixed Methods Appraisal Tool (MMAT) quality appraisal tool was used [52], matching the study design to the quality appraisal tool for evaluating methodological quality and risk of bias. This tool is used across studies widely [53,54]. Based on each study’s fulfillment of the tool’s criteria, a percentage was given based on the quality appraisal. For differing ratings, authors discussed discrepancies until a consensus was reached. Quality percentages ranged from 60 to 100% based on when a study met 1–5 items of the tool’s criteria (see column 6, Table 2). Due to the limited number of studies identified after inclusion/exclusion criteria were applied, studies were not excluded based on quality. These quality assessment tools help identify potential study bias and internal and external threats to validity.

## 3. Summary of Findings

The electronic database search initially yielded 1780 publications (ATLA Religion, 118; CINAHL, 444; PsychInfo, 513; PubMed, 245; SocIndex, 460). After 456 duplicates were removed (see Figure 1, inclusion and exclusion criteria were applied, and grey literature (hand search) revealed one additional study. Following the full-test screening, 12 articles were eligible for acceptance for this integrative review.

Study designs in the final study count of 12 included quantitative (N = 2), qualitative (N = 9), and mixed method (N = 1). Studies were conducted worldwide, with the majority of studies conducted in Europe (N = 5) or North America (N = 4). Some studies did not specify the dementia stage (N = 3), some ranged from mild (N = 2) to severe (N = 2), and some grouped stages (N = 5). Perspectives were collected from a variety of participants: studies included professionals and care staff such as physicians, nurses, care aids, clergy, therapists, housekeeping staff, social workers, etc. (N = 6), PLwDs (N = 3), and others combined professionals, family members including caregivers, and PLwDs (N = 3). The majority of included study settings were nursing homes and long-term care (n = 6). Data were extracted from included studies and synthesized into thematic categories (Table 3).

The included articles defined spirituality as the essence of a person, a search for meaning and life purpose for connectedness with important sources, including self, others, nature, and/or a higher power. Spirituality was associated with religion but was defined as a broader concept. Spiritual needs, as needs for spirituality, were defined as a sense of meaning and life purpose to find peace and well-being through connectedness. Two articles mentioned a theoretical approach [41,47] which included the ethical theory of caring [55] and personhood, and the ethics of dementia care [56,57].

### 3.1. Thematic Domains

#### 3.1.1. Characterizing Spiritual Needs, Preferences, Resources, Approaches, and Support

Five studies explored the theme of characterizing spiritual needs, including preferences, resources, and approaches to and support for spiritual needs [40,41,42,43,48]. Four of these studies were qualitative, and one study used mixed methods. These included studies exploring spiritual needs across the different stages of dementia.

##### In Residents with Dementia, Family, Nursing Home Staff

Two studies focused on the spiritual needs of PLwDs from multiple perspectives, including residents, family members, and nursing home staff [40,41]. Spiritual needs include individual and in-community needs [40]. These are needed to express spirituality and religiosity alone or in a group. Spiritual needs also included the need to participate in religious rituals and practices such as prayer at mealtimes and bedtime, participating in Holy Communion, lighting Sabbath candles, citing religious exclamations, singing and listening to religious songs, holding religious objects, and talking about religious activities (i.e., church choir) [40,41]. Spiritual activities included participating in lifelong hobbies, playing card games, listening to classical music, and enjoying holiday celebrations [41]. From these perspectives and in this particular study, participants expressed that spiritual needs are religious needs. Family members and nursing home staff reported that residents with dementia have spiritual needs of connectedness and need support to nurture the spiritual self within [41]. Resources identified to support the spiritual needs of PLwD included holding spiritual and religious activities and working with spiritual care providers. Nursing home staff emphasized the importance of identifying and accommodating the religious diversity of PLwDs. Spiritual concerns were expressed through prayer and included doubt and disillusionment.

##### In Residents with Dementia

Studies [42,43,44] emphasized the importance of and characterized the spiritual needs of PLwDs. Chen et al. [42] (N = 10) reported that the spiritual needs among community-dwelling older adults with early-stage dementia centered on wishes for reversing impaired memory and loss of independence. In Balqis et al.’s study [43], ten older adults living with mild to moderate dementia in long-term care institutions received various forms of spiritual support, including support to get closer to God or help in worship and support when nearing the end of life. Toivonen et al. [44] emphasized the importance of personalizing spiritual care to cover four identified elements of spirituality: religion, meaningful relationships, nature, and art.

##### In Nurses

Toivonen et al. [45] draw on the experience of 17 nurses who worked in dementia care to understand the spiritual needs of PLwDs. The nurses worked on different specialty units, providing varying perspectives of spiritual needs in different contexts. The nurses all observed that PLwDs expressed spiritual needs through verbal and nonverbal communication, in either direct or indirect form. Direct requests included requests for nurses to assist older adults in performing spiritual activities (i.e., reading the scriptures, praying, etc.). Indirect expressions of spiritual needs were identified in the attribution of tangible objects resulting in physiological changes (i.e., religious images helped PLwDs to calm down).

#### 3.1.2. Characterizing Spiritual Care and Support

##### In Clergy

One study [46] investigated how the clergy meets the needs of Alzheimer’s patients and their families. This qualitative study (n = 12) explored how members of a faith community, specifically the clergy, describe experiences of spiritual connections related to Alzheimer’s disease within Mennonite and Lutheran congregations in Virginia, U.S. The authors identified that clergy discussed the importance of simple religious rituals, such as prayer and song, in helping maintain spiritual connections for individuals with Alzheimer’s disease. However, the authors heard that many participants felt there was a lack of training for clergy and an opportunity to create a curriculum for an educational program for clergy and leaders in faith communities.

#### 3.1.3. Meaning of Spiritual Care

##### In Residents with Dementia, Family, and Staff

One study [47] investigated the meaning of spiritual care for PLwDs. This qualitative study (n = 29) explored the meaning of spiritual care from the perspectives of patients living with moderate to severe dementia, their families, and their care providers within an urban tertiary center in Canada. The authors identified spiritual care as recognizing and attending to little things that promote a sense of personhood, such as being intentional in caring, listening to, and being with patients, and upholding religious rites and rituals.

#### 3.1.4. Assessing Spiritual Care Interventions + Support

Three studies assessed spiritual care interventions and support from different perspectives.

1. In Residents with dementia: Aloustani et al. [48] conducted a randomized control trial to investigate the effect of group spiritual therapy on the cognitive state of 50 older adults aged 60 with mild dementia. The result reported that group spiritual therapy, as a low-cost intervention, significantly enhanced the cognitive state in older adults (*p* < 0.01) over two weeks.
2. In others: Connelly and Moss [49] examined whether music is useful for accessing spiritual needs and providing meaningful spiritual support for PLwDs. This qualitative study (n = 4) utilizing interpretative phenomenological analysis (IPA) explored the experiences of music therapists and chaplains working with PLwDs in hospitals. Authors identified five emerging themes from their analysis: (1) music can facilitate spiritual expression; (2) spirituality is necessarily a broad and evolving term; (3) spirituality may be a coping mechanism for PLwD; (4) music therapy contributes to validating the individuality of PLwD; and (5) collaborative work between music therapy and pastoral care is worthy of further exploration.
3. In nurses: Of importance are the perspectives of healthcare workers who provide direct care to PLwDs. Palmer et al. [50] interviewed healthcare providers (n = 24) with no religious affiliation providing direct care to patients with dementia from Boston, M.A. (i.e., chaplains, nursing staff, social workers, and activities professionals) to gain insight into the spiritual needs of PLwDs. Findings suggest that loss of cognitive capacity may impact older adults’ ability to access faith in dementia, often leading to anxiety, spiritual distress, and frustration. However, there is the possibility that spiritual intervention at the mild stage of dementia may mitigate spiritual needs in severe dementia, per the providers.

#### 3.1.5. Predicting Spiritual Care Provision

##### In Providers

One study examined the provision of spiritual care at the end of life for residents with severe dementia living in long-term care in the Netherlands [51]. Perspectives from physicians who provided end-of-life care for residents with dementia were collected prospectively and retrospectively. Of the 88 physicians participating, only 20.8% provided spiritual care before death. Predictors of spiritual care provision were identified as family satisfaction with physicians’ baseline communication, residents finding spirituality and faith very important, and when residents had female family caregivers.

## 4. Discussion

This review found that the spiritual needs of PLwDs are present across all three stages, reported by multiple perspectives (i.e., PLwDs, family members, providers, spiritual leaders, etc.), universal across the world and religions, essential to support for personhood, and described in various ways. These spiritual needs of PLwDs include needs through everyday interaction and for spiritual and religious rituals and expression. The findings and categories reported above have been compiled into the following discussion points for ease of application and which refer back to our original research questions, “What do we know about the spiritual needs of PLwD?” and “How are spiritual needs addressed for PLwD?”.

### 4.1. Characterizing Spiritual Needs

PLwDs have social and emotional needs, some of which may be better defined as spiritual needs [56,58,59]. Spiritual needs can be *individual* or *in community*. PLwDs may need reminders and resources provided for them to maintain their established spiritual and religious rituals and activities by themselves and in a community with others. As they progress to severe dementia, not all PLwDs may respond to established religious and spiritual resources, but many still do. For many observed and interviewed across these studies, PLwDs’ faith appeared to remain, even after cognition declined. Providers and family reported that PLwDs in the advanced stage had moments of lucidity when exposed to familiar hymns, singing the words and enjoying the music, even for those who appeared past communication. This is similar to other reports supporting the use of religious and spiritual activities with an emotional and procedural component for those with advanced dementia [60].

*Related to religious needs*, spiritual needs are also related to *everyday moments*. Understanding and identifying elements central to each person’s spirituality is key to informing care and support. Holistic patient care practiced by healthcare providers includes a compassionate presence and reflective listening, which promotes well-being and reconciliation [21,61]. In our included studies, participants responded to spiritual and religious rituals and activities with a calming effect. Participants reported it comforted PLwDs and provided a sense of safety through hope and peace, instilling confidence amid loss of control. Other benefits of holistic spiritual care for advanced illness include decreasing depressive symptoms and anxiety and maintaining social relationships [33,35,62]. Holistic care through daily patient engagement honors each unique individual’s values and supports their identity and personhood, which is greatly needed in caring for PLwDs throughout the progression of the condition [63].

The spiritual needs of PLwDs can be expressed through *verbal* and *nonverbal* behavior, are related to past experiences with *meaning*, and prompt *connectedness* through meaningful relationships with others, nature, art, and the significant. Spiritual and religious expressions and rituals reported in the studies included Sabbath candles, mealtime grace, bedtime prayers, religious texts (e.g., the Bible, Torah, and Quron), prayer books, religious symbols (e.g., Rosary, crucifix, cross), individual and group prayer, music, art, nature, holiday celebrations, favorite hobbies, Eucharist, and religious service attendance. Engaging in these activities helps instill some sense of control over one’s current life as PLwDs experience loss of independence and functional decline. Connecting PLwDs with familiar sources of meaning and connection is reaffirming, helping them feel understood and valued. Indeed, PLwDs use spiritual and religious activities for emotional support to help them cope, find a sense of control through embracing their faith in the significant, and find meaning after a dementia diagnosis [64].

### 4.2. Addressing Spiritual Needs through Spiritual Care

Healthcare providers across disciplines, especially in palliative care, can support a PLwD’s spiritual needs by providing access to religious and spiritual rituals and activities for expression, with compassionate presence through everyday interactions, and by honoring their personhood [39,65]. A direct report of spiritual needs from PLwDs in the earlier stages is ideal, though as we have seen here, caregivers, providers, and others closely working with PLwDs have an important perspective, too. Fostering a connection for PLwDs with others and with nature, art, and the significant, healthcare providers display acceptance and reaffirmation through supporting the familiarity of the PLwD’s meaningful sources. One such care program engaging residents with dementia, Namaste Care, is an international, person-centered multisensory program providing meaningful activities and compassionate care to support the needs of the spirit and the body [39,66].

By supporting the identity and spiritual preferences of PLwDs, providers are reaffirming the meaning of their existence. There is an increasing need to provide access to faith activities and rituals for PLwDs. As their progressive loss of cognitive abilities develops, this loss impedes their independent access to spiritual aids. As a result, PLwDs have a more challenging time connecting with those needs, and some may not be able to express their spiritual needs [63,67]. Thus, providing reminders and assistance to available religious and spiritual resources is essential.

### 4.3. Spiritual Care Barriers to Addressing Spiritual Needs

Several barriers were reported by participants in addressing the spiritual needs of PLwDs. These included factors in healthcare protocol, staff limitations, and PLwDs’ health issues. Healthcare barriers included low priority of spiritual care, not being a routine part of care, the staff being too busy, and transportation not being readily available. Staff-related barriers included limited spiritual care competence and experience, lack of understanding, lack of opportunities to learn, lack of administrative support, prioritizing non-dementia residents’ participation over PLwDs’ participation, and doubting the worth of taking PLwDs to religious and spiritual activities. Health-related barriers included behavioral expressions and incontinence of PLwDs, which inhibited staff from taking them to religious and spiritual activities. These barriers are not new but are also reported in other spiritual care provision articles [4,21,68,69].

Spirituality and spiritual care remain increasingly essential subjects within the field of gerontology; questions continue to arise, however, about how they are connected to PLwDs [4,23,70,71]. Person-centered care has been at the forefront of the long-term care scene for many years [72] and continues to grow in popularity, innovation, and implementation [73,74], particularly regarding PLwDs [75,76,77,78]. More studies are needed to assess the spiritual needs of PLwDs and to provide tailored care to support those needs. Specifically, tools are needed for assessing the spiritual needs of PLwDs. In addition, spiritual care interventions targeting commonly reported concerns of PLwDs, such as fear, loss of self, emotional pain, and anxiety around memory loss, are greatly needed. However, without proper education, training, and emphasis placed on spiritual care provision amongst healthcare organizations, barriers will remain for healthcare staff in identifying and supporting these needs for meaning and connection in PLwDs.

As the concept of meaning frequently arose throughout the included studies, healthcare providers could focus on connecting PLwDs to sources of meaning to support their spirituality. As each person’s expression of spiritual needs is individualistic, identifying personal sources of meaning for PLwDs could be a tangible way for healthcare providers to understand and focus on supporting spiritual needs instead of remaining unclear about what spirituality is. Future research could explore the sole concept of meaning, perhaps using the work of Viktor Frankl [78]. Additionally, tailored interventions incorporating meaning should be explored to support PLwDs’ spiritual needs.

## 5. Conclusions

Spirituality plays an essential role in the lives of older adults and is an important factor in health, well-being, and preserved cognitive function as adults age. Despite its importance, there is limited research on the spiritual needs of PLwDs and how to address them. As a dimension of palliative care, spiritual care to address the spiritual needs of PLwDs is an essential component of the bio-psycho-social model of care; more research is needed to investigate the effects of spiritual care for supporting spiritual needs in this population. In addition, studies are needed in the growing population of adults with young-onset dementia. There is a need for studies that address the spiritual experiences of PLwDs, the spiritual resources they use to meet their spiritual needs, and the impact of spirituality on their health and well-being in general. As meaning often arose in the included studies, a literature review focused specifically on meaning alone is warranted and could bring additional depth and enrich this research space. In addition, findings from this integrated review underscore the important role of healthcare, especially of palliative care providers, healthcare educators, and researchers who are committed to promoting the holistic care of PLwDs in ensuring that future healthcare providers, nursing education curricula, and clinical and research work are responsive to the spiritual needs of PLwDs.

## 6. Limitations

There are some limitations to this integrative review. First, publication bias may have affected the findings because the search was limited to peer-reviewed literature. Gray literature, case studies, unpublished reports, dissertations, and articles published in languages other than English were not included. Therefore, relevant studies may have been omitted. Second, the designs of the reviewed studies were primarily surveys or qualitative interviews, and only one study utilized randomized control trials. Third, the measurement and description of dementia stages were inconsistent; four studies did not specify the stage of dementia [45,46,49,50], which limits the comparison of findings. Next, almost all studies were conducted at a long-term care facility. PLwDs who receive care at home may have a different experience. Lastly, future studies could examine cultural context and variation across faith traditions and groups to elucidate differences further.

## Figures and Tables

**Table 1:** Search terms and databases.

Database	Search Strategy
ATLA religion	(dementia OR Alzheimers OR cognitive impairment OR memory loss OR Lewy
	body OR Lewy bodies) AND (needs OR care)
PsychInfo	(dementia OR Alzheimers OR cognitive impairment OR memory loss OR cognitive
	(decline) AND (spirituality OR spiritual needs OR religious needs OR spiritual care OR existential care OR faith)
CINAHL	(dementia OR Alzheimers OR cognitive impairment OR memory loss OR
	cognitive decline OR Lewy body OR Lewy bodies) AND (religious needs OR spiritual needs OR spirituality OR spiritual care OR existential care)
PUBMED	(dementia OR memory loss OR Alzheimer OR cognitive decline) AND (spirituality OR spiritual needs OR religious needs OR spiritual care OR existential care OR faith)
SOCIndex	(dementia OR Alzheimers OR cognitive impairment OR memory loss OR cognitive
	decline OR memory) AND (spirituality OR spiritual needs OR religious needs OR spiritual care OR existential care or faith)

**Table 2:** Included study findings (N = 12).

Authors and Year Published	Study Purpose	Region and Setting	Dementia Type and Stage Evaluated	Study Participants/Data Source	Study Design and Quality Ratings *	Major Relevant Findings
In residents with dementia, family, NH staff						
Schmidt et al., 2018 [40]	To identify, recognize and meet needs of older adults with advanced dementia towards end of life	Germany urban and rural, and religious and non-religious nursing homes	Advanced vascular, Alzheimer’s disease, or unspecified dementia GDS = 6–7 and loss of verbal communication ability	Health professionals (N = 41) working with advanced dementia residents (i.e., caregivers, providers, housekeepers, and others)	Qualitative Grounded theory Semi-structured group discussions, interviews, and observations 5/5 rating	Spiritual needs are religious needs Spiritual needs, either individual or communal: (1) religious expressing, (2) participating in religious rituals including religious exclamations, songs, mealtime prayers, communion, holding religious objects, and talking about religious activities such as church choir or excursions Residents displayed positive reactions and signs of well-being Some previously religious residents did not show interest
Powers and Watson, 2011 [41]	To identify and examine multi-perspective views of residents’ spiritual orientation, practices, preferences; perceptions of spiritual nurturance and support; and resources and approaches to spiritual needs	New York, USA, 48 nursing homes with and without religious affiliations	All stages: mild, moderate, and severe dementia CPS = 0–6	Residents with dementia (N = 83), family members (N = 30), and nursing home staff (N = 66) (clergy, pastoral care providers, nurses, nursing assistants, social workers, recreation and physical therapists, housekeepers, food service workers, administrators, and volunteers) mild (N = 26) moderate (N = 30) severe (N = 27) N = 36 unable to verbally communicate N = 47 able to verbally communicate	Mixed methods concurrent nested, predominantly qualitative 4/5 rating	Residents: Religious practices: Sabbath candles, mealtime grace, and bedtime prayers Spiritual activities: classical music, longtime hobbies, card games, and holiday celebrations Spiritual concerns: prayer, doubt, and disillusionment Family/NH Staff: residents need spiritual connectedness support, and need to nurture spirit within Institutional resources/approaches:religious and spiritual activities held at facilities and spiritual care providers NH Staff: importance of accommodating religious diversity
In residents with dementia						
Chen et al., 2019 [42]	Explore spiritual needs of older adults living with mild dementia in the community	Taiwan, community receiving home care services from mental health hospital staff	Mild dementia CDR = 1 or MMSE 18–23	Older adults living with mild dementia (N = 10) Ages 68–93 years	Qualitative Descriptive, semi-structured interviews with content analysis 5/5 rating	Four themes: (1) desire to turn back time, (2) to retain some control of remaining life, (3) to instill meaning into past experiences, and (4) to rely on faith-based strength Spiritual needs centered around wanting to turn back time and reverse impaired memory and loss of independence
Balqis et al., 2021 [43]	To identify support needs of older adults with dementia living in long-term care	Indonesia, long-term care	Mild to moderate dementia CDR, score unspecified	Older adults living with dementia, (N = 10), mild stage (N = 7), and moderate stage (N = 3) Age 64–86 years High school education or higher	Qualitative Descriptive phenomenology with structural analysis 4/5 rating	Older adults living with mild to moderate dementia have ability to share their experiences for holistic support (i.e., bio-psycho-social-spiritual needs) Spiritual support included support to get closer to God, help in worship, and at end of life Holistic support can help maintain current abilities and improve their quality of life LTC needs to improve the quality of care and quantity of caregivers to maximize holistic support for this population
Toivonen et al., 2023 [44]	To understand how older adults with dementia experience spirituality and spiritual support in nursing care	Finland, home care and long-term care	Various types of dementia, including Alzheimer’s, Vascular, and unspecified Mild to moderate	Older adults living with dementia (N = 10) and family members (N = 9)	Qualitative Ricoeurian hermeneutic phenomenology with structural analysis 5/5 rating	Older adults living with dementia need spiritual support in nursing care, which should be personalized Four elements of spirituality were identified: religion, meaningful relationships, nature, and art Barriers identified in nursing care provision: spiritual care competence, limited time, presence, and experience
In nurses						
Toivenen et al., 2018 [45]	Describe the experiences of nurses supporting spirituality in the care of older people living with dementia	Finland, dementia Nursing units	Unspecified, but includes severe dementia	Female nurses and nursing assistants (N = 17) with at least 1 year of nursing experience	Qualitative Heideggerian hermeneutic phenomenology with inductive analysis 5/5 rating	Spiritual needs of older adults can be understood through verbal and non-verbal expressions, verbally both directly and indirectly, and by valuing their spiritual backgrounds
In clergy						
Tomkins and Sorell, 2008 [46]	To explore how clergy meet the needs of Alzheimer’s disease patients and their families	Virginia, USA, their own established churches and congregations within local communities	Unspecified	Clergy (N = 12) from Mennonite and Lutheran congregations	Qualitative Open-ended interviews and focus groups Grounded Theory 3/5 rating	Many of the clergy stated the importance of simple religious rituals, such as prayer and song, in helping maintain spiritual connections for individuals with Alzheimer’s disease Lack of training for clergy, and an opportunity to create a curriculum for an educational program for clergy and leaders in faith communities
In residents with dementia, family, NH staff						
Carr et al., 2011 [47]	To explore the meaning of spiritual care from the perspectives of patients living with moderate to severe dementia, their families, and their care provider	Canada, urban tertiary care center	Moderate to severe dementia MMSE > 10	(N = 29) Older adults with dementia (N = 8) Family members (N = 5) Healthcare workers (N = 11)	Qualitative Hermeneutic phenomenologicalOpen-ended interviews 5/5 rating	Spiritual care focuses on promoting personhood through intentional caring attitudes and actions: listening to and being with, meeting religious needs, and facilitating religious rites and rituals Recognizing and attending to ‘little things’ promoted a sense of personhood and connectedness to self and others Spiritual care provides opportunities for the one caring to feel valued, cared for, and connected
In residents with dementia						
Aloustani et al., 2021 [48]	Effect of group spiritual therapy on cognitive function of older adults	Iran, center for older adults	Mild dementia MMSE > 20	Older adults with dementia (N = 50), Experimental group (N = 25) and control group (N = 25) Age 60 years and above	Randomized control trial For 2 weeks 4/5 rating	A significant difference in effect of cognitive state after the spiritual therapy intervention (*p* < 0.01) Group spiritual therapy can be used as a complementary, low-cost, and effective method for improving the cognitive state of older adults
In others						
Connelly and Moss, 2021 [49]	Whether music may prove a useful tool for assessing spiritual needs and providing meaningful spiritual support for people with dementia	Ireland, hospital setting	Unspecified	Music therapists (N = 3) and a pastoral care professional (N = 1)	Qualitative Open-ended interviews Interpretative phenomenological analysis 3/5 rating	Five themes: (1) musichas the capacity to facilitate spiritual expression; (2) spirituality is necessarily a broad and evolving term; (3) spirituality may be a coping mechanism for people with dementia; (4) music therapy contributes to validating the individuality of the person with dementia; and (5) collaborative work between music therapy and pastoral care is worthy of further exploration As individual services, music therapy and pastoral care are exploring how to provide good-quality spiritual care for people with dementia
In nurses						
Palmer et al., 2022 [50]	Explore the salient spiritual needs in dementia to inform future intervention development	Boston, USA, community-based and long-term care facilities	All dementia stages (mild, moderate, and severe)	Providers including chaplains (N-10), nursing staff (N = 6), social workers (N = 6), and activity professionals (N = 2)	Qualitative Semi-structured with thematic analysis 5/5 rating	No difference in findings by provider type or by religious/spiritual affiliation Spiritual experience in dementia differs from other medical conditions (1) fear, profound loss of self, inability to access faith, and progressive and incurable nature of dementia make it different (2) there is a window of opportunity in the mild phase since there is awareness of mild dementia, which precipitates spiritual distress
In providers						
van der Steen et al., 2014a [51]	Examine provision of spiritual end-of-life care in dementia	Netherlands, 28 long-term care	All-cause severe dementia BANS-S ≥ 17	Long-term care physicians providing care at end-of-life for residents with dementia (N = 88)	Prospective and Retrospective Unspecified length of time 5/5 rating *	Spiritual end-of-life care was provided shortly before death to 20.8% of the residents Predictors of end-of-life spiritual care provision were families’ satisfaction with physicians’ communication at baseline, faith or spirituality very important to residents, and female family caregiving

Notes. NH = nursing home; LTC = long-term care; CPS = Cognitive Performance Scale; MMSE = Mini-Mental State Examination; CDR = Clinical Dementia Rating; BANS-S = Bedford Alzheimer Nursing Scale—Severity Subscale. * Study quality appraisal was evaluated with the Mixed Methods Appraisal Tool (MMAT) [52].

**Table 3:** Included study characteristics (N = 12).

Characteristics	Frequency (n)
*Publication year*	
2008–2014	4
2015–2020	3
2021–2022	5
*Region*	
the Netherlands	1
USA	3
Canada	1
Germany	1
Iran	1
Taiwan	1
Indonesia	1
Finland	2
Ireland	1
*Setting*	
Long-term care	6
Community-based	3
Hospitals Variety of settings	2 1
*Dementia stage evaluated*	
Unspecified	3
All three stages	2
Mild	2
Mild to Moderate Moderate to Severe Severe	2 1 2
*Study participants/Data source*	
Professionals	6
PLwD	3
Combined	3
*Study design*	
RCT	1
Mixed Methods	1
Qualitative	9
Quantitative	1

*Note.* PLwDs = persons living with dementia; RCT = randomized control trial.

## Data Availability

Materials are available from the corresponding author upon request.
